# Marketing factors associated with a continuous positive airway pressure machine purchasing in patients with obstructive sleep apnea

**DOI:** 10.2144/fsoa-2022-0073

**Published:** 2023-03-23

**Authors:** Bundit Sawunyavisuth, Nattaporn Sopapol, Chi-Hsing Tseng, Kittisak Sawanyawisuth

**Affiliations:** 1 Department of Marketing, Faculty of Business Administration & Accountancy, Khon Kaen University, Khon Kaen, 40002, Thailand; 2 Department of Medicine, Faculty of Medicine, Khon Kaen University, Khon Kaen, 40002, Thailand; 3 Department of Marketing & Distribution Management, National Pingtung University, Pingtung, Taiwan, 900391, Republic of China

**Keywords:** continuous positive airway pressure machine, predictors, purchasing, sleep apnea

## Abstract

**Aim:**

Obstructive sleep apnea (OSA) is related with several cardiovascular diseases. It should be treated with a continuous positive airway pressure (CPAP) machine. There is limited data on marketing factors on a decision of CPAP machine purchasing in OSA patients.

**Materials & methods:**

We enrolled adult patients aged over 18 years with OSA who tried a CPAP. Marketing factors were evaluated for a decision of CPAP machine purchasing.

**Results:**

There were 95 OSA patients participated in the study. Nice color CPAP machine and good knowledge and informative salesperson had adjusted odds ratio (aOR) of 4.480 and 9.478, the other two factors had aOR at 0.102 and 0.217.

**Conclusion:**

Marketing factors related to CPAP machine purchasing in patients with OSA.

## Plain language summary

Marketing factors are crucial for patients or customers to decide of purchasing goods or medical devices. This study shows machine features, machine information via brochure, and knowledge of salesperson/nurses can be associated with a decision of patients with sleep apnea to buy a machine.

## Graphical abstract

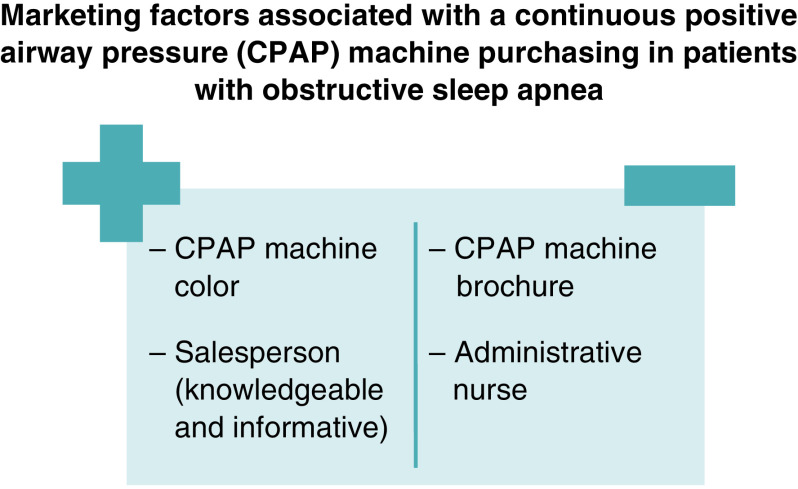

Obstructive sleep apnea (OSA) is characterized by repeated upper airway obstruction during sleep, resulting in intermittent hypoxemia. The diagnosis of OSA is established based on the presence of an apnea–hypopnea index (AHI) of five or more events/hour through polysomnography (PSG) [1]. OSA is very prevalent and has been reported to be related to several diseases, but it may be underdiagnosed. A report from the US estimated that 24 million adults were undiagnosed [2]. In addition to the issue of undiagnosed OSA, it has been estimated that 936,360,689 adults worldwide may have OSA regardless of OSA symptoms [3]. The prevalence of OSA in the general population may be over 50% in several countries such as Brunei (77.2%), Bahrain (73.0%) and France (72.1%) [3]. In the US, the prevalence of OSA was found to be 33.2%, with an estimated 54 million patients, while in China, the prevalence was found to be 23.6-42.88%, with over 175 million patients [3,4]. Additionally, the prevalence of OSA may be even higher in some specific populations. OSA is found in 30–60% of patients with heart failure, 42% of patients with ischemic stroke, 85% of patients with atrial fibrillation [5], 41.89% of patients with hypertension [6] and 57.69% of patients with hypertensive emergencies [7]. A study from China found that the prevalence of OSA in admitted Type 2 diabetes mellitus patients was 60.0% [8], while patients with Type 2 diabetes mellitus who had diabetic nephropathy had an even higher prevalence of OSA, at 88.2% [9].

In addition to its high prevalence, OSA has various risk factors and symptoms. A meta-analysis was conducted on 34 studies from 28 countries, with 37,599 patients in total [10]. This study found that age of over 35 years increased the risk of OSA, with an odds ratio of 4.5 (95% confidence interval of 1.4 to 13.8); BMI of 25 kg/m^2^ or higher had an odds ratio of 3.5 (95% confidence interval of 1.2 to 10.5); and alcoholism had an adjusted odds ratio of 4.5 (95% confidence interval of 1.8 to 11.1). Men are at higher risk of OSA compared with women, with an approximately 5:1 ratio, but this ratio is reduced to 1:1 in women of postmenopausal age. Oropharyngeal factors may also be risk factors of OSA regardless of age or sex. These factors include the Mallampati score, a large tongue, a deep palatal vault, torus palatinus, torus mandibularis, retrognathia and cephalometric measurement [11–14]. Some co-morbid diseases also increase the risk of OSA such as hypothyroidism, chronic kidney disease (CKD), HIV infection, acromegaly, Marfan syndrome, allergic rhinitis, epilepsy, asthma and chronic obstructive airway disease.

The most common symptom of OSA leading to patients seeking medical services is unrefreshing sleep with excessive daytime sleepiness [15]. However, only 50% of patients with OSA in the general population reported excessive daytime sleepiness [15]. This symptom was related to the severity of OSA and the oxygen desaturation index, with odds ratios of 20.27 (95% confidence interval of 1.58 to 26.97) and 4.05 (95% confidence interval of 1.86 to 8.81), respectively [10]. Other reported symptoms of OSA include snoring, dizziness, dry mouth, heart burn, headache, dyspnea, palpitation, insomnia and nocturia [15–17]. Snoring may be found in up to 60% of patients, while nocturia more than two-times/night can be found in 30% [15,16]. The nocturnal gastroesophageal reflux symptom is very common and has a prevalence of up to 75% in patients with OSA [15]. Symptoms of OSA may vary among individuals. Note that some patients with OSA may be asymptomatic [15].

Full-attended PSG is the gold standard laboratory test for OSA. This type of PSG is classified as type 1 and reported as the AHI or respiratory disturbance index (RDI). If the AHI or RDI is five or more events/hour, a diagnosis of OSA can be made [18,19]. There are three other available PSG tests including type 2 or unattended PSG, type 3 or the home sleep apnea test, and type 4 or single-channel PSG. Type 3 PSG is an alternative type of PSG with a sensitivity of 80% and has an increasing trend in clinical practice as it is more cost-effective, reliable, and available compared with full-attended PSG [15,20]. Currently, there are some new technologies available in the market and clinical practice that can be used for the diagnosis of OSA, such as smart watches and arterial tonometry. These technologies also have a good sensitivity of over 90% [21,22]. However, the WatchPAT may over-estimate some sleep parameters including total sleep time, rapid eye movement sleep time, and sleep efficiency [21].

Weakness of the genioglossus muscle results in occlusion or upper airway obstruction in patients with OSA, which is the main pathogenesis of OSA. This occlusion may be expressed as snoring or sleep stridor. Repeated hypoxemia is the main mechanism of OSA, leading to activation of the sympathetic system and inflammatory processes. The major consequences of OSA are cardiovascular diseases. The American Heart Association made a scientific statement that OSA is prevalent in patients with cardiovascular diseases including hypertension, heart failure, coronary artery diseases, pulmonary hypertension, atrial fibrillation, and stroke [23]. The prevalence of OSA in these diseases varies from 40 to 80%. These data may imply two things. First, OSA may have a causal relationship with the mentioned diseases. The early detection of OSA may primarily prevent these cardio-vascular diseases. Second, OSA should be screened in patients who develop these cardiovascular diseases, as the treatment of OSA may diminish or prevent further morbidity. Other than these statements by the American Heart Association, several reports have shown significant associations with other diseases or conditions such as gastroesophageal reflux disease, fatty liver, road accidents, perioperative complications, and sudden death [24].

There are several treatments for patients with OSA including continuous positive airway pressure (CPAP) machines, mandibular advancement devices, weight reduction in obese patients, smoking and alcohol cessation, regular exercise, and sleep hygiene [15]. Note that surgical interventions have a limited role in the treatment of OSA, while a mandibular advancement device is suitable in selected cases. CPAP therapy should be administered as the first-line treatment of OSA patients as CPAP therapy is effective and supported by plenty of scientific data. CPAP therapy leads to health status improvement such as a reduction in blood pressure by 10 mmHg, a reduction in the risk of recurrent atrial fibrillation by 56%, a reduction in stroke mortality by 18%, and a reduction in pulmonary hypertension after 12 weeks of CPAP therapy [5]. Additionally, it has been reported that the quality of life of patients with OSA was significantly improved after CPAP machine titration, which was performed for 1 night in the sleep lab or 3–5 nights at home. The Psychological General Well-Being Index also increased from baseline (69.5 to 74.7; p < 0.0001) [25].

As CPAP therapy has several health benefits and can improve wellbeing, attending physicians tend to encourage patients with OSA to purchase a CPAP machine for their personal use. The CPAP machine purchasing rates in patients with OSA are varied, with an average of 40–50% [26,27]. Previous reports presented several predictors of CPAP machine purchasing in patients with OSA, such as NMI and OSA severity [28,29]. Patients with OSA who had a higher BMI (35.0 vs 31.1 kg/m2) or a more severe condition (AHI 40.77 vs 34.75 events/hour) tended to purchase a CPAP machine compared with those with a lower BMI or a less severe condition. Note that an AHI of 30 or more events/hour is considered severe OSA. In 2022, a meta-analysis of eight articles from four countries, with 1605 patients in total, was published. Among the 11 studied variables, 6 were significantly related to the decision to purchase a CPAP machine, namely, age, education, income, smoking habit, sleepiness scale, and AHI/RDI [30]. These data showed that specific customer factors were associated with CPAP machine purchasing in patients with OSA.

Several marketing factors may affect customer behaviors in purchasing medical devices. A previous study from China found that the price and brand of the device may relate to the purchasing decision [31]. Treatment of OSA is also related to the decision to purchase a device for personal use. Therefore, marketing factors may be a potential factor associated with decision making in patients with OSA. Even though there are several reports on clinical factors associated with CPAP machine purchasing in patients with OSA, there are limited data on the correlations of marketing factors and patient decisions in CPAP machine purchasing. Therefore, this study aimed to evaluate if marketing factors are associated with CPAP machine purchasing in patients with OSA.

## Literature review

### Theoretical Background

There are five steps of consumer decision-making process or five-stage model of the consumer buying process including need recognition, information search, evaluation of alternatives, purchase, and post purchase behavior [32]. When customers have a need or want for purchasing a thing, they will search for information both internally and externally to assist the purchasing decision. An internal information is information from previous or past experiences on using the product, while an external information includes data from personal experiences on the product by friends or relatives and data from public resources. The public resources other than recommendations from friends or relatives are television ads, online review or social media recommendation, online review by someone who customers do not know in real life, in-theater advertising, magazine ads, product placement, reseller/channel partner website, manufacturer or vendor website [32]. The most common source is recommendations from friends or relatives (81% of customers), while television ads was the second most common source (65%) and manufacturer or vendor website was the least common source of information (42%). Then, the customers may have emotional connections or surrender to ads with an aim to find the best deal for the product or evaluation of alternatives step. When the customers have decided to purchase the products, the customers may find where, when, and how to buy the product. Finally, after purchasing, the customers may have post purchase step if they have a match or mismatch with expectation and follow-up activities.

In this present era of technological advancement, Malter *et al.* proposed a concept of update ICABS framework [33]. The ICABS framework comprised of I: information, C: cognitions, A: affect, B: behavior, S: satisfaction. This framework has been modified due to technological advancement during this digital age. For example, the customers may get product information from social media sources or websites; some technologies may impact on customer cognition; or satisfaction has shifted from a personal to a shared experience on online sources.

### Conceptual framework

There are limited data on marketing factors on CPAP machine purchasing. Therefore, we applied general concepts of marketing factors on CPAP machine purchasing including product, price, distribution, promotion, and perceived value [34,35]. A study conducted on consumers' preferences and acceptance of organic food products found that product, price, distribution, and promotion had good indicators of the decision to purchase organic food products. These factors had significant standardized regression coefficients in the model to predict product acceptance. Of these four factors, price and promotion had the highest coefficients at 1, while distribution and product had the coefficients of 0.194 and 0.137, respectively. Note that this survey was conducted among 1051 respondents.

A meta-analysis found that patients with OSA who had high income may have higher chance to purchase CPAP machine than patients with OSA who had low income for 47% (95% confidence interval of 1.06, 2.05) [30]. Another study found that CPAP machine purchasing rate was 55.5% in patients with OSA who had social security system. Those who were seriously ill and had public health insurance coverage often received CPAP machine [27]. Therefore, CPAP machine price may be one factor associated with a chance of CPAP machine purchasing as well as health insurance coverage.

Sales promotion has been shown to be one marketing strategy to enhance product purchasing [35–38]. A quantitative study conducted on 278 customers of retails stores in Malaysia found that sales promotion was significantly associated with purchase decision (p = 0.042) [35]. This study also found that perceived value was significantly related with purchase decision in retail stores with a coefficient of 0.593 (p < 0.05). To imply with patients with CPAP, physicians, nurses, or salesperson may be the one who are able to provide information regarding health benefits of CPAP machine in patients with OSA. Finally, post sale marketing may be associated with patient satisfaction and increase chance of purchase. A study conducted at retail sector in Botswana with 100 participants found that approximately 90% of the customers needed post sale marketing or services [39]. The standardized coefficient for after sales services was 0.083 (p = 0.000) for customer retention and 0.148 (p = 0.000) for customer loyalty. As CPAP machine is a device which required maintenance service after purchase, post sale service such as customer care, or service center may be needed.

## Materials & methods

This was a cross-sectional study conducted at Sleep Clinic, Srinagarind Hospital, Khon Kaen University, Khon Kaen, Thailand. This study was a part of CPAP marketing project. The inclusion criteria were adult patients aged over 18 years, diagnosed as OSA by polysomnography, agreed for CPAP therapy and started on CPAP trial for the first time (naive to CPAP machine), and willing to participate the study. We excluded patients with pregnancy or unable to read/understand Thai. A convenient sampling method to recruit participants from the clinic [40,41]. The study period was between October and December 2018. Diagnosis of OSA was made by evidence of the AHI of 5 events/hour or over. Patients with OSA were advised to perform a CPAP trial or CPAP machine titration for three consecutive nights with an automatic CPAP machine at home. After the CPAP trial, patients decided whether they purchased the CPAP machine for their personal uses or not. The CPAP used for CPAP trial is an auto-CPAP machine by Philips Respironics, USA. The patients were instructed for the CPAP trial by the sleep technician. Data of CPAP trial were downloaded after the completion of the CPAP trial.

All eligible patients were requested to fill out a self-reported, non-validated questionnaire after the CPAP trial. The questionnaire mainly evaluated perceptions of marketing factors [42,43]. It comprised of baseline characteristics, education, income, insurance, co-morbid diseases, and marketing factors. The marketing factors included seven categories: CPAP machine feature (22 items), CPAP machine price (4 items), sale channel (9 items), marketing strategy (5 items), salesperson (10 items), post-sale marketing (2 items), and medical personnel (physicians: 10 items and nurses: 8 items). In each item, the patients were asked to rate how the factor associated with their decisions of CPAP machine purchasing. Some factors may be an indicator of ideal or desired CPAP machine such as safety or effectiveness. The score in each item of marketing factors was a 5-level Likert scale with a maximum score of 5 indicating strongly agreed. Details of the questionnaire are as follows:

- Baseline characteristics of eligible patients included age, sex, weight, height, BMI (kg/m^2^), education (primary school, secondary school, college or higher), income categorized as less than 5000 Baht/month, 5001 to 10,000 Baht/month, and over 10,000 Baht/month, occupation, co-morbid disease, and health insurance for CPAP machine covering.
- CPAP machine feature: CPAP machine quality, imported CPAP machine, safety of CPAP machine, model of CPAP machine, size of CPAP machine, color of CPAP machine, CPAP machine power cord, CPAP tube, CPAP mask, CPAP strap, options of CPAP machine, CPAP machine package, CPAP machine bag for traveling, CPAP machine warranty, how easy to use CPAP, information tag of CPAP machine, understandable instruction, effectiveness of CPAP machine, free trial of CPAP machine, and CPAP machine company.
- CPAP machine price: various CPAP machine price, reasonable CPAP machine price, coverage of CPAP machine by insurance, and negotiable CPAP machine price.
- CPAP machine sale channel: direct sale via salesperson in hospital, via hospital shop, via shops outside hospital, convenient buy process, waiting time, payment options, fast delivery, and home delivery.
- Marketing strategy: advertisement in medical brochure, CPAP machine brochure, CPAP machine advertisement via internet, installment payment for CPAP machine, and installment payment by credit card for CPAP machine.
- Salesperson: personality of salesperson, appropriate dress of salesperson, knowledge of salesperson on CPAP machine, ability to demonstrate CPAP machine use, politeness of salesperson, smiley salesperson, friendly salesperson, good knowledge and informative salesperson, enthusiastic salesperson, and ability to answer questions or concerns regarding CPAP machine.
- Post-sale marketing: availability of call center for CPAP machine and availability of service center for CPAP machine.
- Physicians: knowledge of physicians, personality of physicians, professionalism of physicians, polite physicians, physician–patient relation, enthusiastic physicians, advice on treatment, easy to understand explanation, clear questions and answers for CPAP machine, and appropriate treatment processes.
- Nurses: knowledge of nurses, personality of nurses, professionalism of nurses, polite nurses, nurse-patient relation, enthusiastic nurses, easy to understand explanation, and clear questions and answers for CPAP machine.

Statistical analyses. Patients were categorized into two groups by a decision of CPAP machine purchasing. Descriptive statistics were used to compare differences between both groups. The marketing factors were evaluated if related to CPAP machine purchasing by a stepwise logistic regression analysis: backward elimination method. All marketing factors were put in the model. Those factors with a p value of less than 0.20 were remained in the predictive model. A Hosmer-Lemeshow Chi square test was used to evaluate a goodness of fit of the predictive model. Results were presented as odds ratios and 95% confidence interval (CI). All statistical analyses were calculated by STATA software, version 10.1 (TX, USA).

## Results

There were 95 patients with OSA participated in the study. The mean age (SD) of all patients was 53.86 years (12.88) with an average BMI of 30.30 (SD 5.96) kg/m^2^. Male sex accounted slightly higher than female sex (54 patients; 56.84%). Regarding education level and income, 16 patients (16.84%) had primary school education and income of less than 5,000 Baht or 166.67 (1 USD = 30 Baht). Almost half of patients (42 patients; 44.21%) worked as a government officer. Fourteen patients (14.74%) did not have any co-morbid disease, while the 85.26% had at least one co-morbid disease including diabetes, hypertension, heart failure, cardiac arrhythmia, myocardial infarction, stroke, or gastroesophageal reflux disease. A covered health insurance for CPAP machine was applied for 56 patients (58.95%) as shown in Table 1.

**Table 1.** Baseline characteristics of patients with obstructive sleep apnea participated in the study of a continuous positive airway pressure machine purchasing (n = 95).

Factors	Values
Age, years*	53.86 (12.88)
Male sex	54 (56.84)
BMI, kg/m^2^*	30.30 (5.96)
Education	
Primary school	16 (16.84)
Secondary school	22 (23.16)
College or higher	57 (60.00)
Income, Baht/month	
<5000 Baht	16 (16.84)
5001–10,000	5 (5.26)
>10,001 Baht	74 (77.90)
Occupation	
Government officer	42 (44.21)
Business owner	13 (13.68)
Others	40 (42.11)
Co-morbid disease	
None	14 (14.74)
Hypertension	58 (61.05)
Diabetes	19 (20.00)
Heart failure	1 (1.05)
Cardiac arrhythmia	10 (10.53)
Myocardial infarction	1 (1.05)
Stroke	6 (6.32)
Gastroesophageal reflux disease	28 (29.47)
Health insurance for CPAP machine	56 (58.95)

Data presented as number (percentage) except * indicates mean (standard deviation).

There were 76 patients with OSA (80.00%) purchased CPAP machine after the CPAP trial. Details of marketing factors between both groups were shown in Tables 2–4. There were two significant factors in the marketing strategy between both groups: advertisement in medical brochure and CPAP brochure (Table 3). The purchased CPAP machine group rated these two factors lower than those who did not purchase CPAP machine group (3.01 vs 3.81 p 0.020; and 2.91 vs 3.81 p 0.012).

**Table 2.** Marketing factors associated with a continuous positive airway pressure machine purchasing in patients with obstructive sleep apnea: continuous positive airway pressure machine features.

Marketing factors	Did not purchase CPAP machine (n = 19)	Purchased CPAP machine (n = 76)	p-value
**CPAP machine feature**			
Good CPAP machine quality	4.37 (0.71)	4.24 (0.64)	0.410
Imported CPAP machine from USA	3.81 (0.75)	3.82 (0.88)	0.738
Safe CPAP machine	4.31 (0.70)	4.32 (0.63)	0.990
Modern model	3.93 (0.77)	3.97 (0.81)	0.812
Small size	3.87 (0.88)	3.95 (0.89)	0.579
Nice color CPAP machine	3.25 (0.68)	3.38 (1.01)	0.333
Appropriate power cord	3.75 (1.12)	4.00 (0.76)	0.438
Appropriate CPAP tube	4.06 (1.12)	4.10 (0.76)	0.734
Fitted mask	3.87 (0.80)	3.95 (0.95)	0.525
Flexible CPAP strap, no pain	4.00 (0.81)	4.02 (0.91)	0.764
Several options of CPAP machine	3.81 (0.83)	3.61 (1.08)	0.726
Nice CPAP machine package	3.43 (0.62)	3.31 (1.09)	0.752
Travel CPAP machine bag	3.93 (0.85)	3.87 (0.96)	0.874
Colorful CPAP machine bag	3.56 (0.72)	3.25 (1.13)	0.325
Appropriate warranty period	3.81 (1.16)	3.91 (0.97)	0.873
Easy to use	3.87 (1.20)	4.11 (0.89)	0.578
Informative tag	4.06 (1.06)	4.00 (0.79)	0.490
Easy to understand instruction	4.06 (1.06)	3.81 (0.96)	0.235
Effective machine	3.87 (1.14)	4.14 (0.78)	0.465
Free trial prior to purchase	3.93 (1.06)	4.20 (0.73)	0.461
Well known company	3.68 (1.13)	3.84 (0.87)	0.636
Famous company	3.81 (1.10)	3.92 (0.88)	0.711

Data presented as mean (standard deviation) of a 5-level Likert scale with a maximum score of 5 indicating strongly agreed.

**Table 3.** Marketing factors associated with a continuous positive airway pressure machine purchasing in patients with obstructive sleep apnea: continuous positive airway pressure machine price, sale channel and marketing strategy.

Marketing factors	Did not purchase CPAP machine (n = 19)	Purchased CPAP machine (n = 76)	p-value
**CPAP machine price**			
Various prices	4.12 (1.02)	3.86 (1.02)	0.356
Reasonable price	3.93 (0.99)	3.80 (0.92)	0.630
Covered by insurance	3.93 (1.23)	3.72 (1.28)	0.531
Negotiable price	3.75 (1.34)	3.27 (1.17)	0.112

Sale channel			
Direct sale via salesperson in hospital	4.25 (0.68)	4.05 (0.83)	0.459
Via hospital shop	4.06 (0.77)	3.57 (1.05)	0.104
Via shops outside hospital	3.62 (1.08)	3.01 (1.14)	0.138
Various sale channels	3.87 (0.88)	3.27 (1.16)	0.236
Convenient buy process	3.87 (0.95)	3.77 (0.99)	0.753
No waiting time	3.87 (0.95)	3.84 (0.91)	0.888
Various payment options	4.00 (0.81)	3.97 (0.96)	0.883
Fast delivery	4.00 (0.81)	4.07 (0.88)	0.667
Home delivery	3.75 (1.00)	3.72 (1.07)	0.972

Marketing strategy			
Advertisement in medical brochure	3.81 (0.83)	3.01 (1.27)	0.020
CPAP brochure	3.81 (0.98)	2.91 (1.27)	0.012
Advertisement via internet	3.68 (0.94)	3.07 (1.19)	0.065
Installment payment	3.68 (1.07)	2.97 (1.31)	0.061
Installment payment by credit card	3.68 (1.07)	3.08 (1.33)	0.124

Data presented as mean (standard deviation) of a 5-level Likert scale with a maximum score of 5 indicating strongly agreed.

**Table 4.** Marketing factors associated with a continuous positive airway pressure machine purchasing in patients with obstructive sleep apnea: salesperson, post-sale marketing, and medical personnel.

Marketing factors	Did not purchase CPAP machine (n = 19)	Purchased CPAP machine (n = 76)	p-value
**Salesperson**			
Good personality	3.62 (0.61)	3.51 (0.95)	0.829
Appropriate dress	3.81 (0.65)	3.78 (0.81)	0.966
Good knowledge on product	4.06 (0.77)	4.14 (0.74)	0.698
Able to demonstrate CPAP machine use	4.06 (0.77)	4.17 (0.72)	0.602
Polite	4.06 (0.77)	4.15 (0.77)	0.616
Smiley	4.06 (0.68)	4.08 (0.77)	0.838
Friendly	4.06 (0.77)	4.12 (0.77)	0.716
Good knowledge and informative	4.06 (0.77)	4.14 (0.70)	0.704
Enthusiastic	4.00 (0.73)	4.17 (0.74)	0.359
Be able to answer questions/concerns	4.06 (0.77)	4.12 (0.75)	0.719

Post-sale marketing			
Available call center	3.81 (1.04)	3.51 (1.24)	0.480
Available service center	4.00 (1.09)	3.52 (1.25)	0.170

Medical personnel			
Physicians			
Good knowledge	4.56 (0.72)	4.62 (0.59)	0.880
Good personality	4.25 (0.85)	4.30 (0.78)	0.865
Professional	4.43 (0.72)	4.40 (0.66)	0.762
Polite	4.43 (0.72)	4.48 (0.65)	0.860
Good doctor-patient relation	4.43 (0.72)	4.57 (0.60)	0.525
Enthusiastic	4.43 (0.72)	4.58 (0.62)	0.435
Advice on treatment	4.50 (0.73)	4.61 (0.62)	0.580
Easy to understand explanation	4.50 (0.73)	4.58 (0.67)	0.659
Clear Q & A	4.50 (0.73)	4.58 (0.67)	0.659
Appropriate treatment processes	4.50 (0.73)	4.55 (0.60)	0.907
Nurses			
Knowledge	4.43 (0.81)	4.30 (0.78)	0.454
Personality	4.25 (0.77)	4.14 (0.81)	0.658
Professional	4.37 (0.80)	4.24 (0.69)	0.401
Polite	4.31 (0.79)	4.28 (0.72)	0.832
Good doctor-patient relation	4.37 (0.80)	4.30 (0.72)	0.624
Enthusiastic	4.37 (0.80)	4.32 (0.69)	0.683
Easy to understand explanation	4.31 (0.79)	4.35 (0.70)	0.903
Clear Q & A	4.37 (0.80)	4.34 (0.69)	0.742

Data presented as mean (standard deviation) of a 5-level Likert scale with a maximum score of 5 indicating strongly agreed.

The predictive model for CPAP machine purchasing by the stepwise logistic regression analysis was shown in Table 5. There were 13 remaining factors in six categories in the model. Of those, four factors independently associated with CPAP machine purchasing including nice color CPAP machine, CPAP machine brochure, good knowledge and informative salesperson, and nurse with good knowledge on OSA. Nice color CPAP machine and good knowledge and informative salesperson had adjusted odds ratio of 4.480 and 9.478, while the other two factors had negative adjusted odds ratio at 0.102 and 0.217 (Table 5). The final model had the Hosmer-Lemeshow Chi square of 4.14 (p 0.843) indicating a goodness of fit of the predictive model.

**Table 5.** Marketing factors associated with a continuous positive airway pressure machine purchasing in patients with obstructive sleep apnea by logistic regression analysis.

Factors	Unadjusted odds ratio (95% confidence interval)	Adjusted odds ratio (95% confidence interval)
**CPAP machine feature**		
Nice color CPAP	1.159 (0.659, 2.039)	**4.480 (1.046, 19.176)**
Nice CPAP package	0.887 (0.517, 1.520)	0.359 (0.089, 1.437)
Easy to use	1.277 (0.749, 2.177)	2.902 (0.827, 10.176)

CPAP machine price		
Covered by insurance	0.870 (0.551, 1.374)	2.389 (0.783, 7.283)
Negotiable price	0.702 (0.429, 1.148)	0.361 (0.108, 1.204)

Sale channel		
Direct sale via salesperson in hospital	0.728 (0.354, 1.495)	0.230 (0.039, 1.356)
Via shops outside hospital	0.690 (0.422, 1.129)	0.395 (0.118, 1.320)
No waiting time	0.961 (0.528, 1.750)	3.105 (0.728, 13.249)
Marketing strategies		
CPAP brochure	0.236 (0.312, 0.877)	**0.102 (0.019, 0.536)**

Salesperson		
Good knowledge and informative	1.170 (0.547, 2.504)	**9.478 (1.179, 76.169)**

Post-sale marketing		
Available call center	0.801 (0.491, 1.307)	4.709 (0.948, 23.374)

Medical personnel		
Knowledge: nurse	0.790 (0.380, 1.642)	**0.217 (0.054, 0.873)**

Bold indicates independent factors by multivariate logistic regression analysis.

## Discussion

The CPAP machine purchasing rate in this study was 80.00%. There were two positive correlations and two negative correlations of marketing factors on CPAP machine purchasing in patients with OSA.

As previous reported, CPAP machine treatment in patients with OSA is cost-effective [44]. A scoping review found that CPAP machine treatment gained incremental cost–effectiveness ratios of 16,499 USD with a maximum of 33,119 USD per quality-adjusted life years. Generally, patients with OSA had an acceptance rate for CPAP machine treatment of 48.6–60% [45,46]. The CPAP machine acceptance rate may be higher in some specific population such as Down syndrome (92% after 1 month trial) [47]. The high CPAP purchasing or acceptance rate in this study may be due to high percentage of insurance coverage and population study age. Previous studies found that no insurance coverage may be a reason for not purchasing CPAP machine in 20–53.33% of patients [48,49] as well as those with low income [50]. Those with age of 75 years or more were less likely to purchase CPAP machine than those who were younger significantly (odds ratio of 0.57; p 0.02) [51]. This study had the average age of 53.86 years which may have higher rate of CPAP machine purchasing. However, this study did not have any interventions which may facilitate CPAP machine acceptance and purchasing such as disease awareness education, comfortable titration experience, or short-term home CPAP trial [45]. A study from Singapore found that a 1-month CPAP trial may also increase CPAP adherence to 78.5% [52]. Note that this study had the CPAP trial of only three consecutive nights due to resource-limited setting.

A previous study found that CPAP machine cost and income may be an important marketing factor regarding a decision of CPAP machine purchasing [53]. OSA patients with higher socioeconomic status had higher chance of than those with lower socioeconomic status by 1.23-times (95% CI 1.02, 1.40), while higher income also tended to purchase CPAP machine by 1.19-times than those with lower income (95% CI: 0.99, 1.43). Other two factors were co-payment and CPAP machine cost which may be a barrier of CPAP machine purchasing indicated by a telephone survey and similar to other home healthcare devices [31,53]. There were 40% of patients with OSA who declined to purchase CPAP machine due to its cost. In this study, we did not find a significant score of CPAP machine price between those who purchased and did not purchase CPAP machine (Table 3) as well as insurance coverage. Even though insurance issue was in the final model, it was not statistically significant (adjusted odds ratio of 2.389: 95% CI 0.783, 7.283) as shown in Table 5. As previously reported, only 50% of OSA patients accepted CPAP therapy regardless of cost or insurance [54]. Instead, CPAP machine feature, specifically to color of CPAP machine which may be an attractive factor, was associated with CPAP purchasing by 4.480-times (Table 5). Note that there was no specific color in the questionnaire. CPAP machine feature is one marketing stimuli in the buying processes in the stimulus-response model [55].

A decision of healthcare consumers is complex particularly patients with OSA to decide if they would like to be treated with the CPAP machine [56,57]. As knowledge translation is one of the key factors for patients or healthcare consumers [58], this study found that salesperson and nurse but not physicians had significant roles in patients with OSA to make a decision on CPAP machine purchasing (Table 5). These two persons had different effects on the decision of patients with OSA. Patients with OSA tended to purchase CPAP machine by knowledge of salesperson (adjusted odds ratio of 9.478) but not the nurse (adjusted odds ratio of 0.217). These results may be explained by roles of these two persons in our setting. The salesperson provides details of the CPAP machine directly to the patients including models, price, and other marketing information [56], while nurses in clinic perform on administrative works of the patients without informing any knowledge on CPAP machine to the patients. Additionally, CPAP machine brochure was not needed as the salesperson gave information directly to the patients resulting in negative correlation with the CPAP machine purchasing in the model (Table 5). Note that advertisement in medical brochure was significant by the descriptive statistics (Table 3), it was not an independent factor and not retained in the final model after adjusted by other marketing factors.

Even though this is the first study to evaluate marketing factors on CPAP machine purchasing in patients with OSA, there are some existing limitations. First, not all of marketing factors were evaluated as it may cause long and exhausting questionnaire. Second, some social psychological factors of patients with medical teams were studied, only the relation with nurses was significant. The final predictive model was included only marketing factors. Other factors related with OSA were not studied [59–63]. Data in this study is the first study on marketing factors and CPAP machine purchasing, further studies with a random sampling method are required to confirm the results of this study and add other possible clinical factors predictive of CPAP machine purchasing in OSA patients.

## Conclusion

CPAP machine feature, CPAP machine brochure, and knowledge of salesperson and nurses were marketing factors related with CPAP machine purchasing in patients with OSA. CPAP machine feature and knowledge of salesperson associated with higher chances of CPAP machine purchasing, while CPAP machine brochure and knowledge of nurse had negative correlations with CPAP machine purchasing. CPAP companies may use these data to facilitate CPAP machine purchasing in patients with OSA which may reduce future morbidity and mortality in patients with OSA.

**Summary points**

- There is limited data on correlation of marketing factors and patient decision on continuous positive airway pressure (CPAP) machine purchasing in patients with obstructive sleep apnea (OSA).
- CPAP machine feature and Knowledge of salesperson support CPAP machine purchasing in patients with OSA.
- CPAP machine brochure and nurses did not promote CPAP machine purchasing in patients with OSA.
